# Awake nasotracheal intubation under bronchoscopic guidance and anesthetic management in a patient undergoing excision of an endotracheal mass: A case report

**DOI:** 10.1097/MD.0000000000027734

**Published:** 2021-11-12

**Authors:** Jonghae Kim, Bum Young Park, Jung A. Lim

**Affiliations:** Department of Anesthesiology and Pain Medicine, School of Medicine, Daegu Catholic University, Daegu, Republic of Korea.

**Keywords:** airway management, airway obstruction, bronchoscopy

## Abstract

**Rationale:**

The main challenge facing anesthesiologists during endotracheal mass resection is securing effective airway management during surgery. It is important to select an airway intubation and airway maintenance method according to the patient's condition and the characteristics of the mass.

**Patient concerns:**

A 74-year-old woman with aggravated dyspnea for 1 year was scheduled to undergo endotracheal mass excision under general anesthesia.

**Diagnosis:**

The mass was 4 × 3 × 3 cm ovoid-shaped, and located 4 cm above the carina, occupying 41% of the tracheal lumen in a preoperative chest computed tomography and bronchoscopy.

**Interventions:**

After preparing extracorporeal membrane oxygenation in case of the inability to ventilate and intubate, we attempted awake bronchoscopy-guided nasotracheal intubation using a reinforced endotracheal tube with an inner diameter of 5.5 mm and outer diameter of 7.8 mm after a translaryngeal block. The tube was passed around the mass without resistance and placed right above the carina. With the tube pulled back above the mass, another tube was introduced from the opened trachea below the mass to the right main bronchus. Following the resection of the tracheal portion containing the mass, the posterior wall of the remaining trachea was reconstructed. The tube placed in the right main bronchus was removed and the tube in the upper trachea was introduced right above the carina. The patient's head was kept flexed once the anastomosis of the trachea was completed, and the surgery ended uneventfully.

**Outcomes:**

The mass was confirmed as schwannoma by histopathological finding. The patient was discharged from the hospital on the 6^th^ postoperative day without complication.

**Lessons:**

Awake bronchoscopy-guided intubation is a safe airway management method in patients with an endotracheal mass. Close cooperation between anesthesiologist and surgeon, and preparation for airway management before surgery is essential. It is necessary to establish alternative plans that can be implemented in the case that intubation and ventilation are not possible.

## 1 Introduction

The incidence of primary tracheal mass is extremely rare and the treatment includes surgery, cryotherapy, laser therapy, endobronchial stents and/or radiotherapy depending on the type of mass.^[1]^ Intraoperative airway management for patients with tracheal mass is especially challenging for anesthesiologists when surgical removal is required.

Several techniques for airway management of patients with tracheal mass are being introduced. It is important to identify the characteristics of the lesion through preoperative examination, and to select an anesthetic technique that is available to the patient by communicating with the surgeon. In addition, preparation of alternatives that can be accessed is required if the first airway management method selected fails.

“Cannot intubate, cannot ventilate” one of the most dangerous situations in airway management, can occur in patients undergoing tracheal mass or stenosis surgery, especially after anesthesia induction. Unlike the awake state in which airway patency is dynamically maintained through negative pressure ventilation, when anesthetized, respiratory muscle tone including the pharyngeal muscle is reduced.^[2]^ In addition, obstruction at the lower airway due to the tracheal mass itself makes airway maintenance and adequate ventilation more difficult. This is also the reason why the alternatives to airway maintenance after anesthesia induction are more limited compared to airway management methods applicable to awake patients. In this situation, the previously introduced methods including laryngeal mask airway (LMA)^[3]^ and high frequency jet ventilation^[4]^ could be countermeasures for situations where clinicians cannot intubate, but these methods have limitations in resolving situations involving cannot ventilate. Therefore, airway management with patients awake is more beneficial and has a wider scope of alternatives to failed methods.

We report a case of safe and effective airway management for ventilation as well as intubation by performing awake fiberoptic bronchoscopy-guided nasotracheal intubation in a patient with an endotracheal mass.

## 2 Case report

A 74-year-old female (height 155 cm, weight 64.7 kg) presented to our hospital's department of respiratory medicine with a chief complaint of aggravated dyspnea for 1 year. The dyspnea made it difficult for her to lie in the supine position but was relieved in a lateral decubitus or sitting position. There was wheezing in both lungs on auscultation but no other symptoms or signs, such as fever or tachypnea, were present. The patient was a nonsmoker with a medication history of calcium channel blocker for hypertension, hypoglycemic agents for diabetes mellitus, and statins for hyperlipidemia.

**Figure 1:**
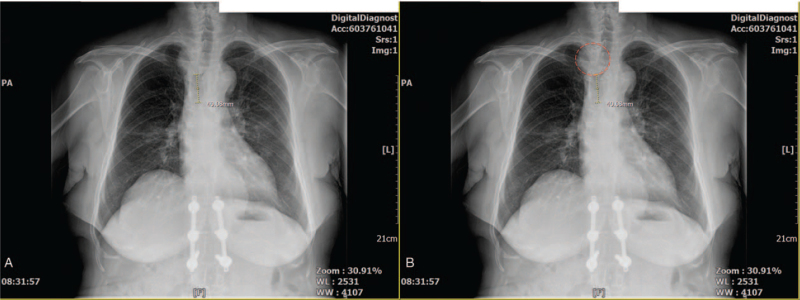
Preoperative posteroanterior chest X-ray showing a tracheal mass compressing the airway 4 cm above the carina (red-dotted circle). To facilitate the identification of the mass that is not clearly visible (A), the red-dashed circle surrounding the mass was placed in the image (B).

**Figure 2:**
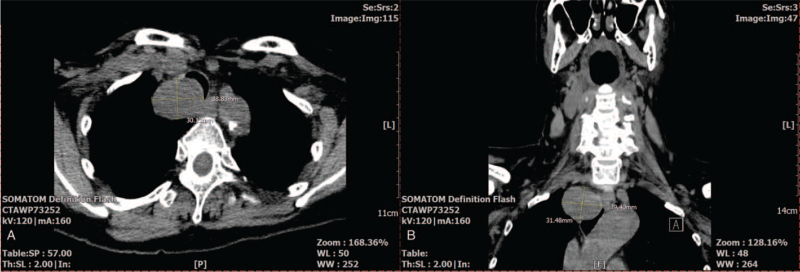
The tracheal mass compressing the airway shown in the axial (A) and coronal view (B) of the preoperative nonenhanced neck computed tomography.

**Figure 3:**
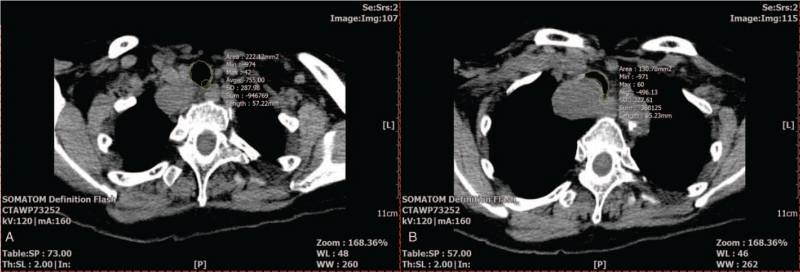
The area of the intact tracheal lumen that was not compressed by the tracheal mass (222.12 mm^2^: A) and the tracheal lumen that was maximally compressed by the mass (130.78 mm^2^: B). The extent of the tracheal lumen compression was calculated as .

**Figure 4:**
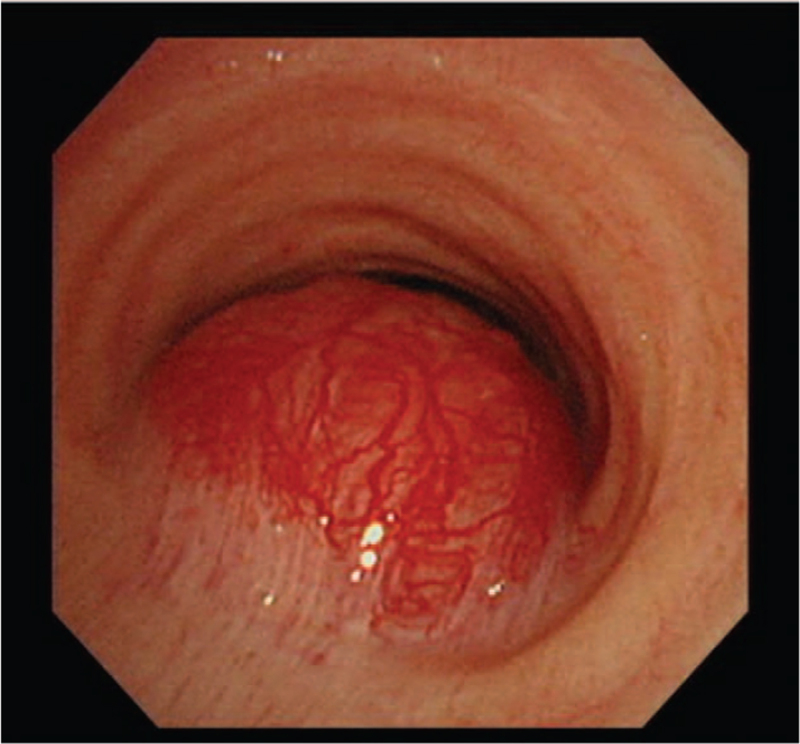
Preoperative bronchoscopy showing a protruding tracheal mass arising from the right posterior tracheal wall.

The arterial blood gas analysis (ABGA) at the time of admission was pH 7.451, partial pressure of arterial carbon dioxide (PaCO_2_) 36 mm Hg, partial pressure of arterial oxygen (PaO_2_) 93.5 mm Hg, and base excess 1.0 mmol/L when 2 L/min of O_2_ were applied via a nasal cannula. The tracheal mass narrowing the tracheal lumen 4 cm above the carina was confirmed in the chest X-ray (Fig. 1A and B). A nonenhanced neck computed tomography (CT) revealed that a 4 × 3 × 3 cm ovoid-shaped mass lesion was abutted to her esophagus in the right paratracheal area (Fig. 2A and B), resulting in approximately 41% luminal narrowing of the trachea (Fig. 3A and B). In a preoperative bronchoscopy, the bronchoscope (BF-260, distal end outer diameter 4.9 mm, Olympus Medical Systems Corp., Tokyo, Japan) was passed around the mass and reached the carina, confirming a right posterior wall protrusion due to the highly vascularized tracheal mass (Fig. 4). The endobronchial ultrasound-guided transbronchial biopsy revealed the mass to be bloody material with hyaline cartilage. It was negative for malignancy. Preoperative pulmonary function tests showed minimal obstructive lung defect with forced vital capacity of 2.36 L (123% predicted), forced expiratory volume in 1 s of 1.62 L (116% predicted), and forced expiratory volume in 1 s/forced vital capacity ratio of 64%. Other laboratory tests showed normal findings.

**Figure 5:**
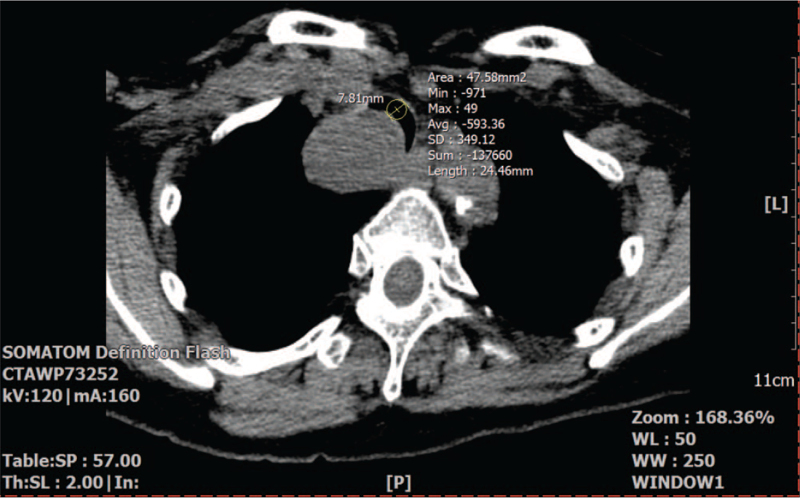
Placement of a circle with a diameter of 7.8 mm in the narrowest tracheal lumen found from the nonenhanced neck computed tomography to simulate the endotracheal intubation using a reinforced endotracheal tube with outer and inner diameters of 7.8 mm and 5.5 mm, respectively.

The patient was scheduled for resection of the tumor with primary anastomosis of the trachea through neck transverse collar incision and a partial upper sternotomy. Based on the results of simulation where an imaginary circle with a diameter of 7.8 mm fitted the narrowest tracheal lumen in the preoperative nonenhanced neck CT (Fig. 5), we planned to use a reinforced endotracheal tube with outer diameter of 7.8 mm and inner diameter of 5.5 mm. The patient's airway was evaluated as Mallampati class III, and her loose teeth (nos. 11, 21) were checked. Considering the possibility of nonventilation after anesthesia induction because of the tracheal narrowing, we decided to use nasotracheal intubation using a flexible fiberoptic bronchoscope while the patient was awake. A set of extracorporeal membrane oxygenation (ECMO) was prepared and remained on standby until the primary control of ventilation. We explained the entire anesthetic procedure to the patient the day before surgery when informed consent was obtained.

The patient entered the operating room in a 30° head-up position while 2 L/min of oxygen were administered via a nasal cannula. All procedures were performed maintaining the same position to minimize patient discomfort. Noninvasive blood pressure, pulse oximetry, electrocardiogram, entropy, and neuromuscular transmission monitoring was applied. After confirmation of initial vital sign [blood pressure 169/84 mm Hg, heart rate 75 bpm, respiratory rate 12 breaths/min, and peripheral saturation of oxygen (SpO_2_) 100%], a modified Allen test was done and an arterial cannula was placed in the right radial artery for continuous blood pressure, cardiac index, and stroke volume variation monitoring using a FloTrac sensor and an EV1000 monitor system (Edward Lifesciences, Irvine, CA). A central venous catheter was placed in the right subclavian vein under ultrasound guidance for central venous pressure monitoring and intraoperative fluid management.

A translaryngeal block using 3 mL of 2% lidocaine was performed while lidocaine pump spray (Xylocaine 10% spray, Aspen, Dublin, Ireland) was applied at the nasopharynx for awake fiberoptic bronchoscopy-guided nasotracheal intubation. The fiberoptic bronchoscope (Ambu aScope 4 Slim, outer diameter 4.2 mm, Ambu, Ballerup, Denmark) was loaded with a reinforced tube (outer diameter of 7.8 mm and inner diameter of 5.5 mm) and inserted via the left nostril. The fiberoptic bronchoscope passed the mass and was placed just above the carina. The tube was carefully pushed through the fiberoptic bronchoscope and placed right above the carina. The tube entered without resistance and there was no bleeding or damage of the mass. After confirming that the patient was breathing through the tube without discomfort, general anesthesia was induced with intravenous propofol and remifentanil using a target controlled infusion pump (Orchestra; Fresenius-Vial, Brezins, France) at target effect-site concentrations (Ce) of 4 μg/mL and 3 ng/mL based on Schnider and Minto pharmacokinetic models, respectively. After loss of consciousness, 60 mg of rocuronium was administered and mechanical ventilation was controlled with 50% oxygen and air. The peak airway pressure was controlled to 22 cmH_2_O with a keeping tidal volume of 5 to 7 mL/kg and end-tidal carbon dioxide concentration (EtCO_2_) of 32 to 39 mm Hg. Anesthesia was maintained with propofol (Ce of 3.0–4.0 μg/mL) and remifentanil (Ce of 1.0–3.0 ng/mL) by keeping the state entropy between 40 and 60.

**Figure 6:**
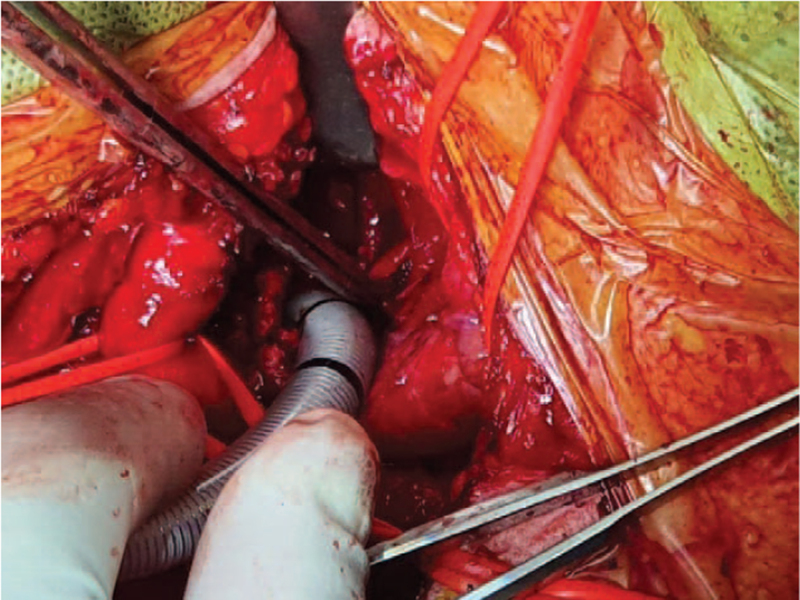
Placement of a reinforced endotracheal tube with an inner diameter of 6.5 mm through the lower trachea into the lumen of the right main bronchus. The cut lumen of the upper trachea was totally clamped. The brachiocephalic vein was displaced downward with a vessel loop to visualize the trachea.

After the patient's head was fully extended by placing a roll under the shoulders, the operation began with a partial sternotomy and trachea exposure in the supine position. The EtCO_2_ was 33 mm Hg with SpO_2_ of 100%. ABGA results were as follows: pH 7.407, PaCO_2_ 40.5 mm Hg, PaO_2_ 180 mm Hg, base excess 0.7 mmol/L. The endotracheal tube was pulled back above the mass and subsequently, the trachea was opened below the mass. Another sterile reinforced tube (inner diameter of 6.5 mm) was introduced through the tracheal lumen until its tip was placed in the lumen of the right main bronchus (Fig. 6). The tube hub was connected through another sterile corrugating tube to the anesthesia machine for temporary one-lung ventilation. Although the peak airway pressure increased to 28 cmH_2_O, the SpO_2_ was maintained at 100%; therefore, the ventilator setting was not changed. The tracheal portion with the mass compressing the right posterior tracheal wall was resected. The anastomosis of the remaining upper and lower tracheal portions started from the posterior tracheal wall. When the posterior half of the tracheal lumen was closed, the reinforced tube placed in the right main bronchus was removed and the endotracheal tube located above the resection region was introduced past the anastomosis site to the carina for two-lung ventilation. The peak airway pressure decreased to 22 cmH_2_O. The results of ABGA were pH 7.380, PaCO_2_ 43 mm Hg, PaO_2_ 171 mm Hg, and base excess 0.2 mmol/L. After confirming that no leakage of the anastomosis site occurred through an air leak test using water and ensuring that there was no decrease in set tidal volume, the wound was closed. To prevent the rupture of the anastomosis site, the patient's head was kept flexed by suturing the chin to the anterior chest. There were no events during the entire perioperative period and at the time of extubation. The patient discharged from the hospital on the 6^th^ postoperative day without complication. The mass was finally confirmed as schwannoma in a histopathological examination. The patient provided the consent for the publication of this case report.

## 3 Discussion

Primary endotracheal tumors are extremely rare and grow progressively. They are often asymptomatic until two-thirds of the tracheal diameter has been occluded, which can lead to a life-threatening condition.^[5]^ However, in our case, although only 41% of the tracheal lumen were occluded, the patient complained of aggravating dyspnea which led to a decision to perform surgery to remove the mass. Intraoperative airway management in patients with an endotracheal mass or severe airway stenosis is one of the most difficult situations encountered by anesthesiologists. Usually, induction of anesthesia predisposes patients to a hypoxic state by decreasing muscle tone, protective reflex, and functional residual capacity. To prevent the hypoxic state, an oropharyngeal or nasopharyngeal airways can be inserted, and manual mask ventilation can be performed. However, if a mass lies in the tracheal lumen, those efforts may be in vain. In such cases, endotracheal intubation needs to be performed to maintain the airway. However, having the endotracheal tube pass the mass may cause bleeding or detachment of the mass debris causing another airway obstruction that could lead to a life-threatening condition, the so called “cannot ventilate, cannot intubate”. In addition, there are fewer back-up options after failed intubation under anesthesia compared to the awake state.^[2,6]^ ECMO may solve the above-mentioned airway problems,^[7]^ but we did not use ECMO as a first option because we were concerned about the complications related to its use, such as heparin-induced bleeding, vascular injury, and limb ischemia.^[8]^ Instead, we had the ECMO on standby, for use in emergency situations.

Alternatively, LMA or high-frequency jet ventilation can be used after anesthesia induction to maintain ventilation and the integrity of the mass to be resected. The use of LMA has the advantage of allowing positive pressure ventilation and oxygenation without disturbing the operation site.^[3,9]^ However, if the LMA does not fit into the patient's larynx structure, the efficiency of ventilation and oxygen delivery to the lower respiratory tract is reduced. In addition, the tracheal mass still exists below the LMA and obstructs the airway. Furthermore, there could be limitations of LMA use if the teeth are loose as in our case. High-frequency jet ventilation also has the advantage of minimizing surgical field movement in tracheal mass resection. However, intermittent EtCO_2_ monitoring is required during ventilation and it is difficult to maintain the desired PaCO_2_. Furthermore, air trap may occur due to a decrease in the efficiency of exhalation in the case of chronic obstructive pulmonary disease or obese patients, with a risk of barotrauma.^[10]^ Therefore, because the use of LMA or high-frequency jet ventilation does not guarantee the maintenance of ventilation after anesthesia induction, we did not use them in our case.

To avoid the events occurring after anesthesia induction without using ECMO, we performed awake fiberoptic bronchoscopy-guided nasotracheal intubation. Although the airway could be maintained while the patient was awake, the possibility of bleeding, displacement, or detachment of the mass during the passage of the endotracheal tube past the mass needed to be reduced. Preoperatively, a bronchoscope with an outer diameter of 4.9 mm successfully passed the mass and reached the carina without compromising the integrity of the mass. In addition, the narrowest lumen of the trachea could hold a circle with a diameter of 7.8 mm corresponding to the size of the endotracheal tube (outer diameter of 7.8 mm and inner diameter of 5.5 mm) in the preoperative neck CT (Fig. 5), which is very close to the range of an appropriate tube size (outer diameter of 8.4 mm and inner diameter of 6.0 ± 0.5 mm)^[11]^ calculated by inputting the patient's height (155 cm) and coronal subcricoid tracheal diameter (13.5 mm according to the preoperative neck CT) in the normogram for the tube size selection.^[12]^ We also expected that using a small-sized tube would facilitate its insertion through the larynx^[13]^ and cause less trauma to the glottis and trachea, particularly in women.^[14,15]^ Hence, we decided to use a flexible fiberoptic bronchoscope with an outer diameter of 4.2 mm, which was loaded with an endotracheal tube with an outer diameter of 7.8 mm and an inner diameter of 5.5 mm. However, because the trachea can have a narrower lumen than the lumen found to be the narrowest in the CT scan, resistance may be encountered while introducing the tube with an outer diameter of 7.8 mm. Nonetheless, we continued to use the tube because the flexibility and nonstatic nature of the trachea allows for tracheal dilatation enabling the insertion of a tube larger than the narrowed tracheal diameter.^[16]^ Accordingly, after the endotracheal tube passed around the mass, we could confirm that the patency of the tracheal lumen and the ventilation through the tube were maintained without any damage to the mass.

The narrower the inner diameter of the tube, the higher the peak airway pressure and the greater the risk regarding the inability to deliver sufficient tidal volume. Although we used the endotracheal tube with an inner diameter of 5.5 mm that was smaller than the tube size (6 mm) causing minimal changes in ventilator pressure,^[17]^ the peak airway pressure was maintained at 22 cmH_2_O, which was clinically acceptable. Because the lower trachea was only 4 cm below the mass, the tip of the endotracheal tube (inner diameter of 6.5 mm) introduced through the lower trachea needed to be placed in the right main bronchus for proper placement of its cuff inside the airway. Fortunately, the patient underwent the one-lung ventilation uneventfully with clinically acceptable airway pressure (28 cmH_2_O).^[18]^

There are several options and methods reported when intubation fails in patients with a tracheal mass (that we did not experience in our case). Saroa et al^[19]^ reported a case of intraoperative airway maintenance in a patient with a lower tracheal mass using a microlaryngeal surgery tube. The microlaryngeal surgery tube, an alternative to the conventional tube in cases where endotracheal tubes do not pass the mass, is longer (32 cm length and 5.0 mm inner diameter), and softer than the conventional endotracheal tube, making it possible to reach the carina while minimizing damage to the endotracheal mass. Another cause of difficulty in intubation is the tube railroading failure due to endotracheal tube impingement in the epiglottis and arytenoid cartilage during bronchoscopy-guided nasotracheal intubation.^[20,21]^ Using a smaller tube or a tube rotation 90° counterclockwise could reduce the intubation failure rate.^[22]^ Warming the tube to make it more flexible could also be a way to better facilitate intubation.^[13]^

In conclusion, we recommend the awake bronchoscopy-guided intubation technique in the presence of tracheal mass. It should be preceded by a thorough understanding of the mass pattern via preoperative examination. Close cooperation between the anesthesiologist and surgeon during intubation and the perioperative period is essential. In addition, other alternatives must be planned and thoroughly prepared in the case that intubation and ventilation fail.

## Author contributions

**Conceptualization:** Jonghae Kim.

**Data curation:** Bum Young Park.

**Writing – original draft:** Jonghae Kim, Bum Young Park, Jung A. Lim.

**Writing – review & editing:** Jonghae Kim, Jung A. Lim.
